# Fluphenazine-Induced Neurotoxicity with Acute Almost Transient Parkinsonism and Permanent Memory Loss: Lessons from a Case Report

**DOI:** 10.3390/ijms24032968

**Published:** 2023-02-03

**Authors:** Roberto De Masi, Stefania Orlando, Vincenzo Toni, Maria Carmela Costa

**Affiliations:** 1 Complex Operative Unit of Neurology, “F. Ferrari” Hospital, Casarano, 73042 Lecce, Italy; 2 Laboratory of Neuroproteomics, Multiple Sclerosis Centre, “F. Ferrari” Hospital, Casarano, 73042 Lecce, Italy; 3 Complex Operative Unit of Ophthalmology, “V. Fazzi” Hospital, 73100 Lecce, Italy

**Keywords:** parkinsonism, Fluphenazine, neurotoxicity, neuroleptics, extrapyramidal syndrome, memory loss

## Abstract

We report the singular case of a 31-year-old woman who developed very serious Fluphenazine-induced parkinsonism over a few days due to a doubly incongruent drug prescription by indication and dosage having been applied to a healthy subject over one week instead of seven months. Unlike gradual drug-induced parkinsonism, our patient experienced acute extrapyramidal syndrome (EPS), reaching significant motor and sphincter disability in just a few days, followed by a gradual incomplete recovery over more than six months. In fact, after drug discontinuation, hypomimia and slight left hemi-somatic rigidity with bradykinesia remained, as well as stable non-progressive memory disturbances. Despite bio-humoral and instrumental investigations and DaTScan were negative, MRI post-analysis evidenced a 6.5% loss in brain volume. Specifically, irreversible cortical and sub-cortical grey matter reduction and cerebrospinal fluid space enlargement with spared white matter were found. Our observations suggest that the sudden availability of Fluphenazine results in a kind of plateau effect of parkinsonism presentation, partially reversible due to the neurotoxic drug effect on the cortical and sub-cortical grey matter, resulting in asymmetric EPS and stable memory loss, respectively. Our report confirms the debated neurotoxicity of first-generation neuroleptics and the postulated theory of differential susceptibility to the cytotoxic stressors on the central nervous system.

## 1. Introduction

Neuroleptic-induced parkinsonism (NIP) is the second-most common etiology of movement disorder in the elderly after Parkinson’s disease [1], and the EUROPARKINSON Collaborative Study estimated that NIP contributes to 5% of all cases of parkinsonian syndrome in Europe [2]. Clinically defined by the 1992 UK Brain Bank criteria as a bilateral symmetric bradykinetic disorder that also includes at least rigidity, rest tremor, or postural instability [3], NIP remains pathologically elusive. Several autopsy cases of NIP patients in fact showed no significant alterations in the central dopaminergic system, consisting of the mesolimbic, mesocortical, tubero-infundibular, and nigro-striatal pathways [1,4].

The mesolimbic–mesocortical pathway connects the ventral tegmentum to the prefrontal cortex, allowing for normal cognitive function, cognitive control, motivation, and emotional response, as well as reward and acquired behaviors such as learned fear and threat conditioning [5,6].

This pathway is thought to function abnormally in psychosis and schizophrenia and also represents the site of action for neuroleptic drugs [7]. However, the dopaminergic system responsible for NIP is the nigro-striatal pathway.

Genetically, DRD1, DRD2, DRD3, and DRD4 have been proposed as HLA genes conferring individual susceptibility to developing extrapyramidal syndrome (EPS) when neuroleptic therapy is started [8]. From the pathophysiological point of view, NIP is thought to be related to drug-induced changes in the basal ganglia motor circuit, secondary to post-synaptic dopaminergic receptor blockade, resulting in significant effects on the firing of Dopamine (DA) systems and, ultimately, on patients’ daily living limitations [9]. The latter are usually reversible, but in 10% of cases the changes can cause irreversible, complete or incomplete EPS due to dopamine receptor blocking agent (DRBA)-induced damage and, in turn, neuronal death [9]. From the molecular point of view, phenothiazines (which are the scaffold of Fluphenazine and other drugs with antipsychotic properties) are thought to target drug efflux pumps such as ABCB1 and P-glycoprotein, as well as AKT and Wnt [10]. The latter two are parallel pathways regulated by D2 receptor antagonists, which are, in turn, involved in the antipsychotic effect. Recent studies have also found the potential of the molecule to reduce the viability of human cancer cell lines, fragment DNA, stimulate apoptosis, and inhibit cell migration and invasiveness in experimental models [11,12].

In this context, we describe a case of Fluphenazine-induced parkinsonism, which is singular because it occurred in a healthy young woman. It is also interesting for its unusually severe clinical characteristics due to the enormous drug dosage administered in seven days instead of seven months using a depot formulation. These conditions constitute a kind of involuntary in vivo experiment on the mechanism of action (MOA) of neuroleptics and their long-standing or permanent effects on the central nervous system (CNS), depending on interaction with D2 post-synaptic receptors expressed in the different neuronal circuits of the nigro-striatal or extra-nigral systems. In fact, the safety and neurotoxicity attributed to mostly first-generation neuroleptics has been a debated topic in the literature for several years. On the other hand, differential susceptibility to cytotoxic stressors on the CNS has also been postulated, but clinical demonstration of this is currently lacking. Therefore, due to its particular and singular conditions, this case report is useful to clarify and deepen our knowledge on these topics.

## 2. Case Presentation

### 2.1. Clinical Assessment

**Figure 1:**
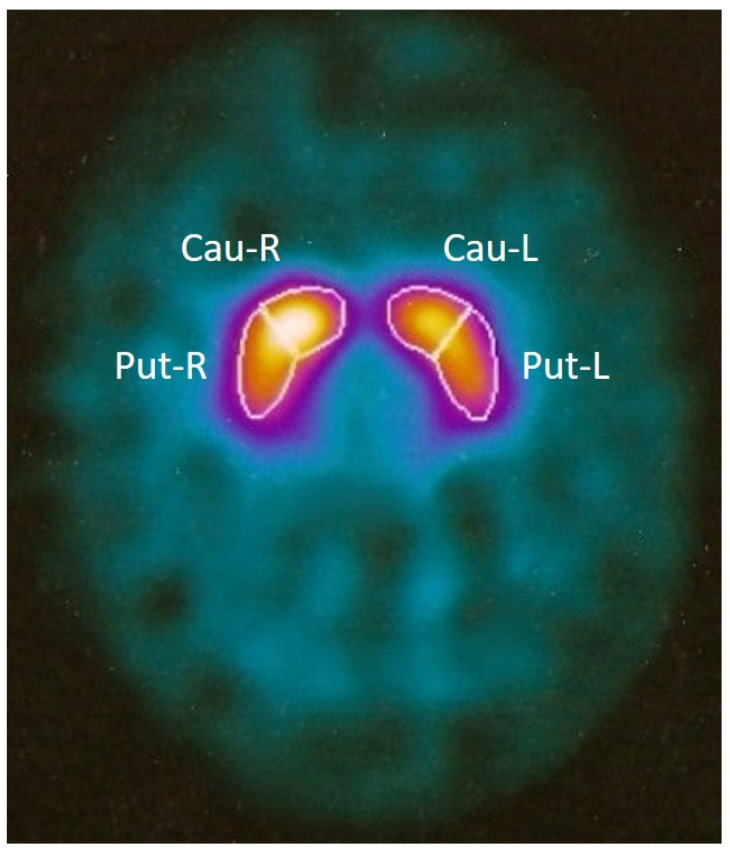
DaTScan assessment. No abnormalities of the nigro-striatal pathway were found bilaterally. Cau-R/L, Caudatum Right/Left; Put-R/L, Putamen Right/Left.

In 2007, a 31-year-old woman, E.B., was admitted to the Complex Operative Unit of Neurology at the “F. Ferrari” Hospital in Casarano (Lecce, Italy) because she had developed very serious parkinsonism over just a few days. Two weeks before admission, the patient had been suffering from a respiratory tract infection, and a pulmonologist had prescribed cephalosporin (Cefonicid—Modiem, VECCHI & PIAM, Anatomical Therapeutic Chemical Classification System Code (ATC): J01DC06) combined with Montelukast and Sobrerol. Since the age of 6, the patient had suffered epileptic seizures, which were treated with Sodium Valproate at a dosage of 500 mg twice a day. However, the patient continued to have three to four crises per year. Any previous significant medications, toxic exposures, and further pathological events were excluded. On admission, neurological examination showed drowsiness, severe apathy, a decrease in spontaneous speech, hypophonia, amimia, severe bradykinesia, limb and axial rigidity with trunk flexion, standing and gait impossible because of falling backwards, resting tremor of hands, and sphincter incontinence. She was unable to attend to any daily life activities. Routine blood tests and tests for HIV, syphilis, and rheumatic diseases were performed. Auto-antibodies, standard cerebrospinal fluid examination, and tau protein level were normal, and 14-3-3 protein was absent. Brain magnetic resonance imaging (MRI) and electroencephalogram (EEG) were normal; 123-Iodine-labelled N-(3-fluoropropyl)-2β-carbomethoxy-3β-(4-iodophenyl) nortropane (123I-FP-CIT) single photon emission computerized tomography (SPECT) imaging, also known DaTScan, showed no abnormalities of the nigro-striatal pathway (Figure 1).

Levodopa 500 mg/day and Amantadine 200 mg/day were started. Sodium Valproate, which can cause parkinsonism [13], was replaced with Topiramate. After hospital discharge, an intensive rehabilitative program was also initiated, as well as a neurological follow-up. Two months later, the patient was still bedridden due to severe parkinsonism of the rigid-akinetic type; resting tremor in upper limbs and moderate apathy were also noted. On UPDRS part III, which ranges from 0 (normal) to 56 (severely affected), she scored 42/56. The Levodopa therapy had given no benefit. Additionally, antibodies against neuronal calcium and potassium channels were negative. The diagnosis of secondary parkinsonism with unrecognized cause was made, until an incredible clerical error casually emerged: “Moditen Depot, 25 mg/day for one week” instead of “Modien” on the general physician’s prescription. Therefore, the patient had been given 175 mg of Moditen Depot (prolonged-release) Fluphenazine Decanoate (FZD) over seven days (more than 0.3 mg/kg per day). Even two months later, FZD and its main metabolite, Fluphenazine sulfoxide, were found in the patient’s urine, but not in the serum. After then diagnosing “Inappropriate administration of Fluphenazine Decanoate-induced parkinsonism”, therapy with Biperiden 4 mg/day, Trihexyphenidyl 6 mg/day, and vitamin E 800 U.I./day was started. Just two to three days later, the patient’s rigidity was slightly reduced, and the other parkinsonian symptoms progressively improved over a period of more than 6 months. Two years later, the patient was autonomous in daily activities and had restarted her job. However, mental examination showed mild loss of memory and apathy, and neurological examination showed slight rigidity and bradykinesia in the left limbs. She was still taking Trihexyphenidyl 4 mg/day.

After a long period of absence from follow-up, the patient was re-evaluated in 2019 at a clinic where she had gone for other reasons. She underwent an MRI as well as a clinical evaluation. Both neurological and neuropsychological examinations confirmed the already known deficits as unchanged.

### 2.2. MRI Assessment

**Figure 2:**
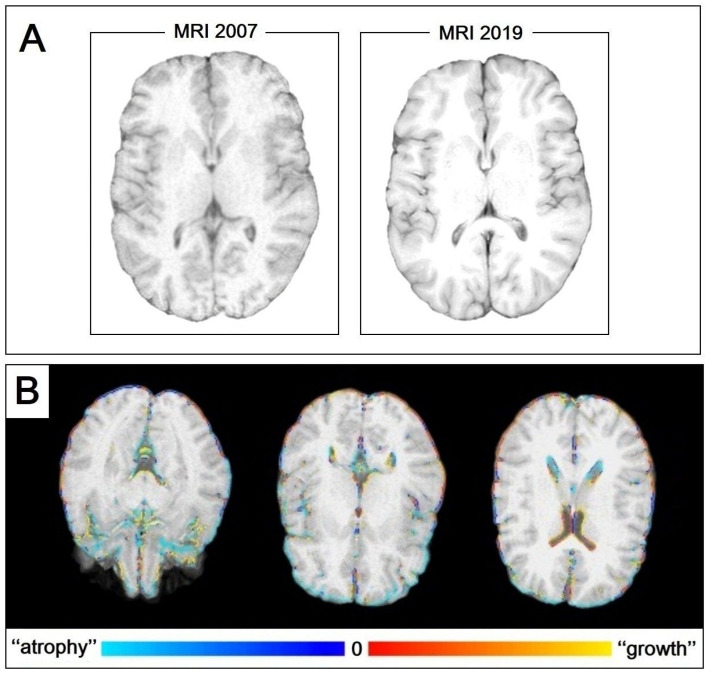
MRI post-analysis. (**A**) T1-weighted axial images of the brain as segmentation output obtained using the Sienax tool. From this comparison of the 2007 and 2019 MRIs, note the structures that most contribute to brain atrophy are the periencephalic liquoral spaces (with a reduction of grey matter and its peripheral fraction). (**B**) Siena color-coded image output of the brain. Blue indicates atrophy; red indicates growth. Note the prevalence of blue in the grey matter, mainly in the peripheral grey matter as well as the ventricular system.

Both the 2007 and 2019 brain MRIs were judged “normal”, but we also performed a post-analysis assessment (Figure 2).

Specifically, we evaluated volumes of grey matter (GM), its peripheral fraction (pGM), white matter (WM), cerebrospinal fluid (CSF), and its ventricular fraction (vCSF) at the two time points using the Sienax tool, and the longitudinal total brain volume (TBV) change using the Siena tool. Both tools are part of the FLS software package created by the Analysis Group (FMRIB, Oxford, UK) according to the voxel-based morphometry methodology. We estimated an initial TBV of 1242.18 mL with an overall reduction of 6.5% over time. GM underwent a 2.15% reduction with an increase of 22.57% in CSF, starting from 589.09 mL and 204.15 mL, respectively. Surprisingly, WM exhibited an increase in volume of 1.77%, starting from 617.71 mL. Furthermore, pGM volume decreased by 7.3%, from 461.82 mL. Finally, vCSF also showed a surprising 21.4% reduction, starting from 23.90 mL. These findings are summarized in Table 1. Written informed consent has recently been obtained as it was only after a long period of time that the patient recovered the motivational conditions to participate in the study.

## 3. Discussion

We have described an unpublished history characterized by permanent EPS secondary to the acute onset of severe parkinsonism due to the erroneous administration of a very high FZD dose in a healthy young subject and in depot formulation. These are to be understood as the best experimental conditions for the in vivo study of the pathophysiology of NIP. In fact, FZD therapeutic doses usually range from 25 to 50 mg every four weeks and accidental FZD overdose has rarely been reported [14]. FZD is a lipophilic molecule that remains in the brain for long periods, and its release is slow. It can cause EPS because it probably exerts its psychotropic actions by blocking pre- and mostly post-synaptic dopamine receptors localized in the central dopaminergic system [15]. In 70 schizophrenic patients treated with Fluphenazine 20–40 mg/day for 10 weeks, parkinsonism was noted in 34%, akathisia in 18%, and dystonia in 36% [16]. Furthermore, a linear relationship between Fluphenazine dosage and EPS in 53 psychotic patients treated with doses of 10, 20, and 30 mg daily was found by Levinson and coworkers [17], but not confirmed by Koreen’s studies [18]. Hassin-Baer and co-authors [19] first suggested the possibility of individual susceptibility to the DA-receptors blockade.

Our patient was undergoing Valproate therapy, which may have played per se a role in determining her parkinsonism by increasing the FZD effects. Specifically, although Valproate-induced parkinsonism (VIP) is widely accepted as a nosological entity, it far from represents a potential confounding factor due to its largely unverified physiopathological mechanisms in the present case. The first Valproate-induced molecular mechanism involves the GABA-dependent inhibition of dopaminergic activity in the substantia nigra and excessive GABAergic activity in the globus pallidus externus, resulting in the over-activity of the basal ganglia pathway [20,21]. Valproate is, in fact, a strong GABAergic effector [22]. This mechanism is widely restored by L-Dopa intake leading to patient improvement, unlike in our case.

Another VIP-postulated mechanism is its direct toxicity on the presynaptic dopaminergic system, with an unmasking effect on sub-clinical dopaminergic degeneration [23]. However, this effect is known to be DaTScan sensitive in nature, unlike the observations of our patient and in the published literature [24]. Finally, enhanced dopaminergic system neurodegeneration due to a mitochondrial dysfunction in complex I of the respiratory chain [25] appears to be the most realistic hypothesis to explain the Valproate involvement in our patient. In summary, the susceptibility of dopaminergic neurons already subjected to Valproate, rather than the high dose of Fluphenazine, may explain the increased axonal loss and related irreversible disturbances.

Unlike the onset of parkinsonism in our patient, which was very early and sub-acute, drug-induced parkinsonism (DIP) is generally gradual and progressive over days or weeks, usually developing within 1–3 months of sustained therapy [9]. Moreover, severe parkinsonism, as in our patient, is rarely reported. The young woman in the study reported by Cheung and Yu [26] was accidentally given FZD 50 mg every four hours, and the error was discovered on the sixth day. At the beginning, she appeared childish and cheerful, but four weeks later she developed parkinsonism. The clinical symptoms spontaneously recovered in one month [26]. Indeed, neuroleptic-induced parkinsonism often resolves within a few weeks after drug discontinuation, although resolution sometimes takes up to a few months [27].

On the contrary, the clinical picture of our patient did not improve over more than 6 months, despite Levodopa (ineffective) and anti-cholinergic (slightly effective) therapies. A possible neurophysiological explanation of these non-conformities may lie in the rapid saturation kinetics at the D2 post-synaptic receptors of the central dopaminergic system, resulting in a plateau effect of the clinical signs presentation, with a steep ascent in the first days and a slow descent over the following 6 months. This explanation seems the most reliable, since it agrees with Levinson [17] and suggests that the likelihood of parkinsonism is dose related. Obviously, the elevated cerebral half-life of the drug is responsible for the long-term D2 inhibition, resulting in slow or incomplete functional recovery. D2 receptors have been studied more intensively because they have a 10- to 100-fold greater affinity for DA than those of the D1 family [28]. Further, the recent fast-off theory suggests that a rapid dissociation from D2 receptors can explain their lower risk of EPS [29,30].

Studies on the molecular physiology of D2 receptor inhibition are elusive, but the ineffectiveness of L-Dopa in our patient suggests stable damage to the dopaminergic system, with its imbalance partially restored by trihexyphenidyl-induced increased cholinergic tone. In fact, the cholinergic interneurons of the basal ganglia can receive an important DA co-release input during burst firing, expressing the receptor D5 (D1-like) [31]. The latter is responsible for an excitatory response after a release burst of DA and D2-like receptors, which finally trigger a hyperpolarization when activated [31].

Autopsy studies have confirmed that some patients who recover from DIP after discontinuing the offending drug have pathological findings characteristic of preclinical PD with evolutive EPS [1,4]. Although the suggestion of neuroleptic induced neurotoxicity is attractive, this finding does not apply to our case. Our patient experienced EPS and memory loss as non-evolving disturbances in both cases. The structural correlation of these is suggested by some evaluations deriving from the MRI post-analysis.

First, a 6.5% brain atrophy over 12 years is too high a value compared to that expected in the general population, with a rate of 0.05–0.3% per year [32]. This inconsistency is explained by the toxic effect of FZD, which acutely exerted a neurodegenerative effect that was partially reversible. The described clinical plateau, followed by the slow and progressive but incomplete recovery, is consistent with acute and partially reversible brain atrophy. As highlighted in Figure 2, the structures that most contribute to brain atrophy are sub-cortical.

Second and as expected, this pathogenic noxa affected all brain structures, inducing GM and pGM atrophy and CSF enlargement. However, WM increased in volume and vCSF reduced, suggesting a partial recovery from the Wallerian degeneration [33]. The resulting net effect on brain volume change evidences a reduction in GM and pGM fractions, as well as a partial restoration of WM and coherent reduction in vCSF. Finally, cortical and sub-cortical involvement can explain residual disturbances, such as parkinsonism and memory loss.

Currently, the molecular mechanisms of neurotoxicity of older-generation antipsychotics, including Fluphenazine, are believed to fall into several major categories: apoptosis, necrosis, decreased cell viability, inhibition of cell growth, increased caspase activity (the “death spiral”), impaired glutamate transport, and mitochondrial damage [34]. These mechanisms are drug-induced in nature and affect the viability of neuronal circuits depending on their time period of action and susceptibility.

## 4. Conclusions

In conclusion, although pathophysiological mechanisms other than dopaminergic neuronal loss in the nigro-striatal pathway cannot be excluded as concomitants, our report confirms the debated neurotoxicity of first-generation neuroleptics, particularly of Fluphenazine, and the postulated differential susceptibility theory to cytotoxic stressors on the central dopaminergic system.

In particular, the unusually high dosage of Fluphenazine, as well as its application in such a short time, suggest the receptorial explanation for the plateau effect of parkinsonism clinical signs due to a lipophilic and decanoate formulation of the drug. Although largely reversible, these signs are partially permanent due to the neurodegenerative effect of the molecule, which affects the viability of dopaminergic neurons and the entire brain tissue. This effect was enhanced by Valproate, directed primarily to the GM and the sub-cortical GM and partially sparing the WM, finally resulting in the enlargement of the ventricular system. These findings on Fluphenazine improve our knowledge of the physiopathology of neuroleptic-induced parkinsonism.

## Figures and Tables

**Table 1:** Brain volumes from MRI post-analysis.

	MRI 2007	MRI 2019
GM	589.09	576.43
WM	617.71	628.64
CSF	204.15	250.23
TBV	1242.18	1161.44
pGM	461.82	428.11
vCSF	23.90	18.79

Brain volumes (mL) resulting from MRI post-analysis in 2007 and 2019 assessments. Note the CSF and WM enlargement in 2019 compared to 2007. On the contrary, GM, TBV, pGM, and vCSF underwent a detectable reduction in the same time period. This finding is in accordance with the abiotrophic phenomenon affecting all brain structures, particularly the GM and sub-cortical GM, with a relative sparing of the WM. GM, grey matter; WM, white matter; CSF, cerebrospinal fluid; TBV, total brain volume; pGM, peripheral grey matter; vCSF, ventricular cerebrospinal fluid.

## Data Availability

Not applicable.
